# Respiratory viruses and the inflammasome: The double-edged sword of inflammation

**DOI:** 10.1371/journal.ppat.1011014

**Published:** 2022-12-29

**Authors:** Kody A. Waldstein, Steven M. Varga

**Affiliations:** 1 Interdisciplinary Graduate Program in Immunology, University of Iowa, Iowa City, Iowa, United Stated of America; 2 Department of Microbiology and Immunology, University of Iowa, Iowa City, Iowa, United Stated of America; 3 Department of Pathology, University of Iowa, Iowa City, Iowa, United Stated of America; University of Iowa, UNITED STATES

## Alveolar macrophage sensing of viral infection

Individuals actively infected with respiratory viruses, such as influenza A virus (IAV), respiratory syncytial virus (RSV), and coronaviruses, transmit by shedding droplets containing live virus while coughing, sneezing, or talking. Respiratory viruses subsequently enter the airways of a host either by coming in direct contact with aerosolized droplets or by an interaction with fomites [1]. The majority of respiratory virus infections are contained to the upper airways and self-limiting; however, lower respiratory tract infections (LRTIs) are a significant cause of morbidity and mortality, especially in children and the elderly [2,3]. When infiltration into the lower airways occurs, infectious viral particles encounter lung resident alveolar macrophages (AMϕ). AMϕ are self-renewing fetal-derived sentinel cells present in the airways that are tethered to the lung epithelium through αvβ6 integrin-latent TGF-β binding [4,5]. Upon direct interaction with viral particles, pro-inflammatory cytokines, or pattern/danger-associated molecular patterns (PAMPs/DAMPs), AMϕ detach from the respiratory epithelium resulting in the induction of increased effector functions and phagocytosis as well as the up-regulation of type I interferons (IFN), chemokines, and pro-inflammatory cytokines [6–8].

AMϕ express a myriad of pattern recognition receptors, including multiple toll-like receptors (TLRs), NOD-like receptors (NLRs), and RIG-I-like receptors (RLRs) that sense viral genetic material and proteins driving NF-κB activation, type I IFN production, and the release of pro-inflammatory cytokines [9]. Early during infection, virus utilization of TLRs as attachment receptors, such as TLR4, as well as other molecules with signaling capacity have been shown to drive innate activation [10]. Intracellular sensors responding to the viral genome including MDA5/RIG-I and TLRs 3, 7, and 8, also mediate activation signals [11]. This early “danger alarm” response by AMϕ is critical to the initiation of the innate immune response within the lung resulting in increased neutrophil and mononuclear cell infiltration and the induction of the antiviral IFN response pathways [6–8]. In vivo depletion of AMϕ resulted in significantly increased disease and mortality in IAV-infected mice [7]. AMϕ activation and pro-inflammatory cytokine production played a critical role in the activation of the immune system and RSV infection. Clodronate liposome depletion of AMϕ prior to infection significantly reduced RSV-induced TNF, IL-6, IFNα, and chemokine production in the lung [8]. AMϕ-depleted mice also exhibited significantly increased RSV-induced airway hyperreactivity as compared to controls [12]. Interestingly, AMϕ significantly contributed to the induction of severe disease and immunopathology following human metapneumovirus (hMPV) infection. AMϕ depletion ameliorated hMPV-induced disease and reduced lung inflammation suggesting excess pro-inflammatory responses contribute to disease [12]. Additionally, AMϕ are essential to the resolution of inflammation and return to a homeostatic state without which severe immunopathology and lung damage can occur [13]. This highlights the multifaceted ability of AMϕ to protect the respiratory epithelium and initiate the innate immune response while also playing an essential role in tissue damage control.

## The inflammasomes

AMϕ highly express large multimeric cellular sensors that reside in the cytosol known as inflammasomes. Canonical inflammasome activation involves 2 signals: Signal 1 is downstream of either TLR or cytokine receptor signaling that induces the activation of NF-κB and the up-regulation of inactive pro-IL-1β and inflammasome components. Signal 2 initiates the oligomerization of the inflammasome complex mediated through direct or adapter protein-mediated (Asc) interaction with the caspase activation and recruitment domain (CARD) of inactive pro-caspase-1 [14,15]. Formation of the inflammasome complex activates and directs caspase-1 enzymatic activity that can then cleave pro-IL-1β/18 to its secreted and mature form [16]. Caspase-1 and -11 can also cleave and activate gasdermin D that forms pores in the cellular membrane. This serves to release IL-1β/18 as well as drive inflammatory pyroptotic cell death [17]. Dendritic cells (DCs) within the lungs and the respiratory epithelium also express inflammasomes and are important in the induction of the immune response to infection and wound healing [18]. However, due to the potency of the inflammatory cascade elicited by inflammasomes, they must be tightly regulated.

Signal 2 activators are unique to each inflammasome allowing specific responses to a variety of stimuli. Canonically, NLRP1 is activated directly by toxoplasma and pathogen proteases, NLPR3 by many cellular stressors converging on cytosolic ion flux, NLRC4 by bacterial protein interactions such as flagellin, and AIM2 by both excess host and pathogen cytoplasmic dsDNA [14–16,19]. However, viruses have been shown to elicit activation of each of the major inflammasomes. Recently, the NLRP1 inflammasome was shown to directly sense cytosolic viral dsRNA and oligomerize in primary human keratinocytes [20]. Both NLRP3 and AIM2 have been widely shown to respond to viral infection and significantly contribute to both viral clearance and the induction of severe disease [21,22]. NLRC4 inflammasome-deficient mice exhibited significantly increased FasL expression on DC following IAV infection. The up-regulation of FasL expression on DC led to reduced CD4 T cell responses by inducing cell death resulting in decreased viral clearance and survival [23].

The NLRP3 inflammasome responds to a wide variety of cellular insults and danger signals including increased levels of reactive oxygen species (ROS), ATP, crystalline particulate matter, pore-forming toxins, and viral protein components [24–27]. Despite extensive investigation, identification of a single mediator of NLRP3 activation by which all stimuli converge remains debated. However, it has been established that binding of NLRP3 and NIMA-related kinase (NEK) 7 is indispensable for the initiation of NLRP3 inflammasome oligomerization [28]. The C-terminal lobe of NEK7 directly binds within the curved leucine-rich-repeat and NACHT domains of inactive NLRP3 “licensing” NLRP3 for activation and oligomerization [29]. This interaction is enhanced by inflammasome priming and signal 2 stimuli and has been proposed to be downstream of potassium (K^+^) efflux [28,29]. Though respiratory viral infection can drive NLRP3 signal 2 by inducing an overall state of cellular stress, more recently, it has been appreciated that pore-forming viral proteins, known as viroporins, can activate the inflammasome. Many respiratory viruses including IAV, RSV, and coronavirus express viroporins that can embed within cellular membranes and facilitate Ca^2+^ and K^+^ ion flux initiating NLRP3 inflammasome activation [26,27,30]. Severe acute respiratory syndrome (SARS) coronavirus (SARS-CoV) expresses 2 viroporins: the E protein and 3a protein [30]. These proteins form pore complexes within cellular lipid bilayers, driving K^+^ efflux and promoting viral replication and daughter virion release [31]. As a consequence of membrane integrity loss, the SARS-CoV 3a protein induces K^+^ ion changes and mitochondrial ROS release activating NLPR3 and IL-1β release [30]. The IAV M2 and the RSV SH proteins act in a similar fashion to promote replication while activating NLRP3 and initiating antiviral immunity.

Following IAV infection, expression and activation of the NLRP3 inflammasome was critical for both innate and adaptive immune responses. Deletion of NLRP3 significantly reduced IL-1β production and recruitment of inflammatory monocytes and neutrophils to the lungs of IAV-infected mice. This correlated with increased viral titer and significant lung damage suggesting NLRP3 inflammasome activation is critical to reduce respiratory viral infection-induced morbidity and mortality [22,32,33]. Additionally, the NLRP3 inflammasome is known to mediate wound healing in the lung through the production of inflammatory mediators and recruitment of macrophages [34]. NLRP3-deficient mice displayed delayed repair of IAV infection driven lung injury and increased collagen deposition [33]. This suggests a role for the inflammasome in both the initiation and resolution of inflammation during respiratory virus infection.

## IL-1 in antiviral adaptive immunity

IL-1β is a potent pro-inflammatory cytokine and primarily associated with innate immunity. Nearly all pathogens can induce IL-1β directly through PAMP recognition by TLRs or through indirect cytokine signaling and self-perpetuation [35]. Under sterile conditions, genetic deletion of IL-1β does not cause spontaneous disease, and upon administration of inflammation inducing adjuvants, IL-1β-deficient mice exhibit reduced febrile responses and decreased IL-6 production highlighting the importance of IL-1β in the inflammatory cascade [36]. IL-1 receptor (IL-R) signaling also induces IFN-stimulated gene expression in infected cells that was essential to restrict viral replication in primary human tissues infected ex vivo [37]. However, more recently, it has been appreciated that IL-1β plays a key role in the promotion of adaptive immunity. IL-1β is known to promote the induction of IL-17 producing CD4 T cells [38]. Others have identified that IL-1β significantly increases the expansion of effector and memory CD4 T cells in response to antigen [39]. Interestingly, IL-1β not only increased the proportion of IL-17 producing CD4 T cells in vivo but also enhanced expansion of Th1 and Th2 cells primed in vitro suggesting IL-1 signaling plays a role in Th17 differentiation and also expansion of all effector CD4 T cell subsets [39]. Professional antigen presenting (APC) cells also respond strongly to IL-1R signaling enhancing their activation and up-regulating antigen processing and cytokine production. This has a direct effect on priming and activation of both CD4 and CD8 T cell responses in vivo [40,41]. Additionally, IL-1R-deficient mice exhibit significantly decreased numbers of CD103^+^ DCs. Upon infection with IAV, DCs within the lung displayed lower levels of maturation markers and a reduced ability to migrate to the draining lymph nodes and prime adaptive responses [41].

IL-1R signaling also directly impacts CD8 T cell responses and promotes memory formation [42]. Interestingly, many viruses express proteins known to inhibit IL-1β through multiple mechanisms including the secretion of soluble decoy receptors. These viral factors led to impaired CD8 T cell memory responses and viral clearance suggesting viruses have evolved to specifically evade enhanced T cell function through IL-1R signaling [43]. These findings have prompted the use of IL-1β as a vaccine adjuvant to boost antigen-specific memory formation [44,45]. Mucosal immunization against IAV with IL-1β as an adjuvant displayed significantly increased IgG, IgG1, and IgG2a antibody levels as compared to 23 other interleukins. Antibodies were detectible in the serum, saliva, nasal wash, fecal extract, and vaginal wash and showed evidence of strong IAV hemagglutinin neutralization capacity. IL-1β-adjuvanted mice exhibited 100% survival and minimal weight loss compared to controls and mice immunized with IL-1α and IL-33 [45]. An RSV adenoviral vector vaccine expressing IL-1β as an adjuvant also displayed significantly increased RSV-specific IgG and IgA production as compared to controls and an IFN-inducing adjuvant. Mice immunized with IL-1β as an adjuvant also exhibited enhanced memory responses and a higher total number of RSV-specific tissue resident memory CD8 T cells within the lung that correlated with increased viral clearance [44]. These findings highlight the potential for IL-1R signaling in modulating adaptive immune responses beyond the canonical role of IL-1 family cytokines in mediating early viral restriction and innate immunity.

## Excessive inflammasome activation drives immunopathology

Though inflammasomes play an integral role in immunity, aberrant inflammasome activation is associated with many severe conditions including lung injury and fibrosis [46]. In the lung, K^+^ efflux and NLRP3 inflammasome activation drives ventilator-induced lung injury, a common treatment in patients with significantly reduced lung function following severe respiratory infection [47]. Mutations in inflammasome components resulting in unrestrained activation have also been shown to play a role in initiating macrophage activation syndrome (MAS) and cytophagocytosis. This induces excessive IL-1R signaling that perpetuates itself driving an autocrine inflammation loop. These patients present with severe systemic inflammation causing recurrent fever cycles, splenomegaly, chronic anemia, and leucopenia. MAS and inflammasome-associated cytokines have been reported in the lungs of fatal SARS patients and were major determinates of lethal H1N1 and 1918 IAV infection [48–50].

In juvenile mice, sustained NLRP3 activation following IAV infection resulted in excessive inflammatory monocyte recruitment and lung pathology that could not be prevented by blockade of IL-1R signaling [51]. These results indicate that the NLRP3 inflammasome contributes to severe disease in IAV infection. However, NLRP3-deficient mice also display significant lung damage following infection and reduced IL-1β production [32,33]. Chemical inhibition of NLRP3 in vivo revealed that early in infection, NLRP3 inflammasome activation is protective and indispensable to control IAV replication and lung pathology. In contrast, later during infection, NLRP3 activation and production of IL-1β contributes to the induction of the cytokine storm and severe immunopathology [22]. This emphasizes the necessity of tightly regulated inflammatory responses in the lung. In hospitalized children with RSV infection, high levels of IL-1β are positively correlated with severe disease and negative clinical outcomes [52]. Similar to IAV infection, inhibition of NLRP3 inflammasome activation reduced RSV-mediated immunopathology and airway disease in mice [53]. Additionally, infants with severe RSV-induced disease exhibit increased levels of the Th17 cytokine, IL-17A, in the upper respiratory tract [54]. In the animal model, RSV-induced neutrophilia, mucus production, and airway hyperresponsiveness, hallmarks of severe disease, were IL-17 dependent [55]. Differentiation of Th17 CD4 T cells in the lung is driven by IL-1β and IL-6, along with IL-23 and TGF-β [38]. This supports a role for the inflammasome in driving pathogenic Th17 responses following respiratory viral infections.

## Conclusions

**Fig 1:**
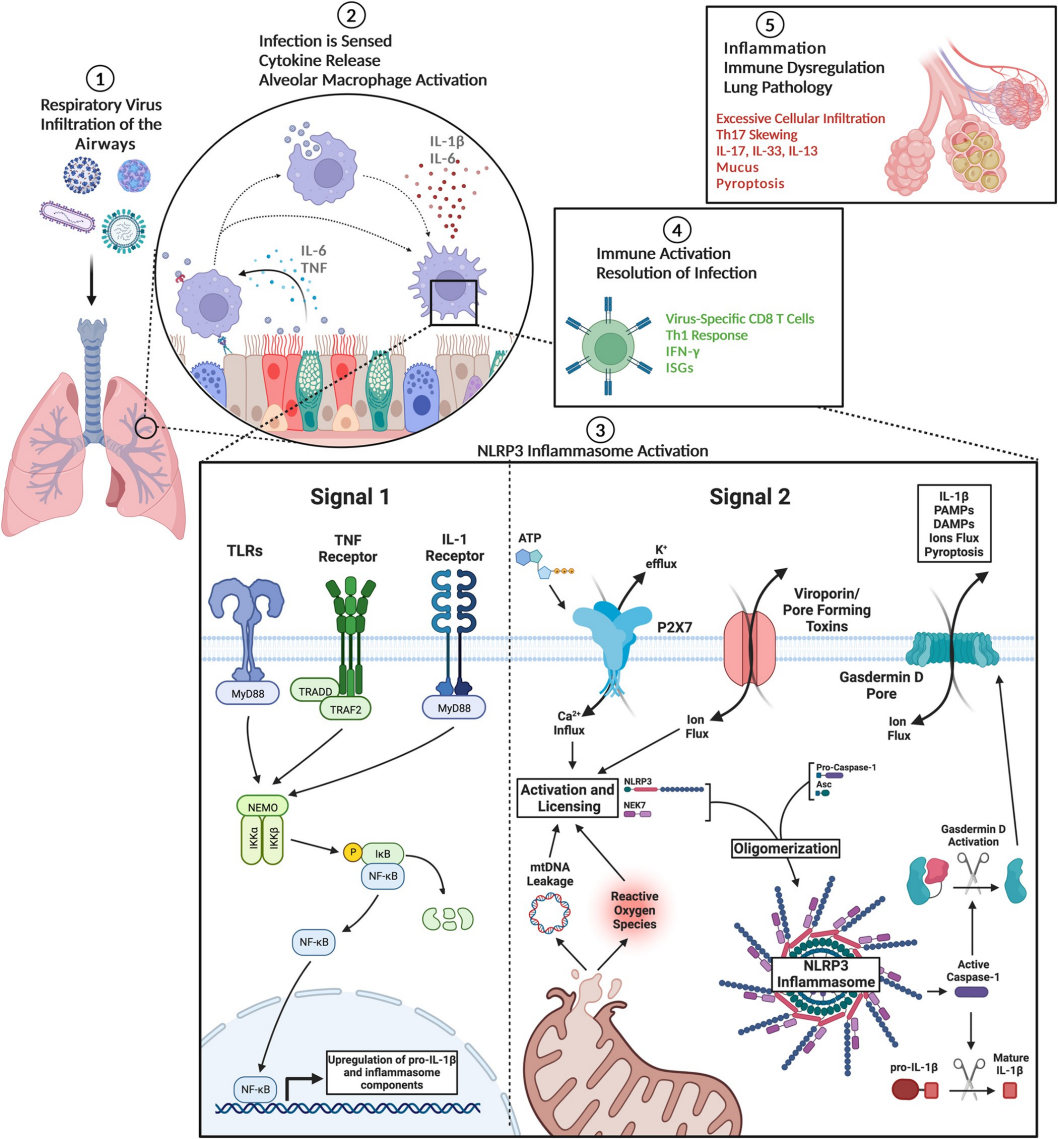
Respiratory virus activation of the NLRP3 Inflammasome. Respiratory viruses enter the airways and come into contact with the respiratory epithelium and alveolar macrophages **(1)**. Infection and/or recognition of pathogen-associated molecular patterns expressed by the virus activate the innate immune response driving the release of pro-inflammatory cytokines such as IL-1β **(2)**. Processing and secretion of IL-1β requires activation of large multimeric sensors known as the inflammasomes. NLRP3 inflammasome priming requires signal 1 activation of NF-κB driving the up-regulation of pro-IL-1β. Signal 2 activation of NLRP3 senses overall cellular stress resulting in changes in cytosolic ion concentration and formation of reactive oxygen species. This initiates binding of NLRP3 with NEK7 that licenses the complex and facilitates oligomerization with the adapter protein, Asc, and inactive pro-caspase-1. Formation of the complex produces cleaved and enzymatically active caspase-1 that can subsequently cleave and activate IL-1β as well as gasdermin D that forms membrane pores leading to pyroptosis **(3)**. Activation of the inflammasome is essential for an effective and robust immune response against respiratory viruses **(4)**. The potency of inflammasome activation can be a “double-edged sword” when left unrestrained leading to severe immunopathology **(5)**. Graphics are the authors’ original work created with BioRender.com.

Discovered over 20 years ago by Dr. Jurg Tschopp and colleagues, the inflammasomes continue to be an area of significant scientific exploration as new roles are continually being observed [16]. During viral infection, the inflammasomes mediate strong pro-inflammatory responses that are important to innate and adaptive immune activation [22,32,33]. Despite their strong capacity to induce inflammation, the inflammasomes also play a key role in lung wound healing and inflammation resolution [33,34]. Specifically, the NLRP3 inflammasome is known to activate following viral infection and respond to a variety of stimuli including sensing changes in cytosolic ion concentrations. Respiratory viruses directly modulate cellular ion flux activate the NLRP3 inflammasome through the expressing of viroporins that facilitate signal 2 activation [26,27,30]. The role of inflammasomes in early viral restriction has been heavily investigated. However, more recently, the role of IL-1R signaling in initiating the adaptive immune response has been explored. IL-1 family cytokines have been shown to increase T cell activation and memory formation leading to their use as experimental vaccine adjuvants [44,45]. Though the inflammasomes play a key role in antiviral immunity, their activation is also strongly associated with immunopathology in the lung (Fig 1). Recent studies have indicated that early inflammasome activation is essential to viral clearance and the reduction of infection-induced disease. In contrast, excessive inflammasome activation later during infection is associated with lung tissue damage and immunopathology, suggesting a temporal role for the inflammasome [22]. Together, these findings demonstrate the “double-edged sword” of inflammasome activation in critical tissues, such as the lungs, where clearance of pathogens without excessive inflammation is essential to reducing morbidity and mortality.
